# Can physical activity levels and relationships with energy expenditure change the clinical aspects of sarcopenia and perceptions of falls among elderly women? Observational cross-sectional study

**DOI:** 10.1590/1516-3180.2020.0602.R1.0402021

**Published:** 2021-05-10

**Authors:** Vitório Luís Kemp, Leonardo de Souza Piber, Ana Paula Ribeiro

**Affiliations:** I MD, MSc. Orthopedic and Sport Medicine Physician, Postgraduate Health Science Department, Medical School, Universidade Santo Amaro (UNISA), São Paulo (SP), Brazil.; II MD, MSc. Professor, Medical School, Universidade Santo Amaro (UNISA), São Paulo (SP), Brazil; and Attending Physician at Department of Radiology and Imaging, Center for Diagnostic Medicine, São Paulo (SP), Brazil.; III MD, PhD. Professor and Coordinator of the Biomechanics and Musculoskeletal Rehabilitation Laboratory, Postgraduate Health Science Department, Medical School, Universidade Santo Amaro (UNISA), São Paulo (SP), Brazil; and Postdoctoral Student, Medical School, Universidade de São Paulo (USP), São Paulo (SP), Brazil.

**Keywords:** Exercise, Sarcopenia, Aged, Women, Physical activity, Elderly, Performance, Fall, Energy expenditure

## Abstract

**BACKGROUND:**

Physical activity (PA) is an effective strategy for managing sarcopenia in the elderly, but few studies have addressed PA levels regarding age-related changes.

**OBJECTIVE:**

To ascertain the effects of elderly women’s PA levels on sarcopenia, physical performance, handgrip strength and perception of the risk of falling, and their relationship with energy expenditure.

**DESIGN AND SETTING:**

Observational cross-sectional study conducted in the southern region of the city of São Paulo, Brazil.

**METHODS:**

Forty-seven elderly women were evaluated and divided into three groups: low PA (n = 13); moderate PA (n = 16); and high PA (n = 18). Their PA levels were investigated through the International Physical Activity Questionnaire (IPAQ); sarcopenia index, through dual-energy radiological absorptiometry; physical performance through the Timed Up & Go test; handgrip strength, using a digital dynamometer; and perception of the risk of falling, through the Fall Risk Awareness Questionnaire.

**RESULTS:**

High PA level indicated higher skeletal muscle mass index, physical performance and IPAQ score, compared with low and moderate PA levels. Multiple linear regression analysis showed that higher IPAQ energy expenditure at high and moderate PA levels was a good predictor of higher physical performance and increased perception of the risk of falling.

**CONCLUSION:**

Elderly women classified as having high PA level showed improvements in sarcopenia, handgrip strength, physical performance and perception of the risk of falling. The IPAQ energy expenditure of the elderly women with high and moderate PA levels was a good predictor of physical performance and improved perception of the risk of falling.

## INTRODUCTION

A sedentary lifestyle is the primary risk factor for muscle weakness.^1^ Sarcopenia, defined as the loss of muscle mass and function, affects quality of life and increases the risk of physical limitations and disability among older adults.^1^^,^^2^ Currently, epidemiological statistics show that sarcopenia affects about 50% of elderly people, depending on the country, ethnicity of the patient, diagnostic criteria and healthcare setting.^1^^,^^2^^,^^3^^,^^4^ Overall, the prevalence among men is about 25% and among women, 20%.^2^^,^^5^ Thus, loss of muscle mass appears to be an inevitable part of the aging process, especially with decreasing physical performance with aging.^5^^,^^6^^,^^7^^,^^8^^,^^9^ Scientific evidence has shown that sarcopenia begins to develop between the ages of 30 and 40 years. It progresses with muscle mass declining by an average of 8% per decade between the ages of 40 and 70 years, and accelerates to 15% per decade from the age of 70 years onwards, with variation between men and women.^8^^,^^10^

Sarcopenia is associated with functional decline, falls, disabilities and fractures, increased mortality among older adults and increased medical expenditure.^1^^,^^2^^,^^3^^,^^4^^,^^5^ This disease has many causes, including genetics, nutritional deficiencies, metabolic disturbances, inflammation through production of proinflammatory cytokines by adipose tissue, physical inactivity^4^^,^^11^^,^^12^^,^^13^^,^^14^ and physical limitations.^15^^,^^16^^,^^17^^,^^18^ Among all these risk factors, research has shown that the greatest cause of sarcopenia is insufficient physical activity and lack of exercise with advancing age, and this has attracted the attention of science and healthcare professionals who are involved in its treatment.^4^^,^^5^^,^^12^^,^^13^^,^^14^^,^^15^

Physical activity (PA) and exercise training have great value in maintenance and augmentation of muscle strength. Increased physical performance enables prevention or treatment of sarcopenia among elderly people.^14^^,^^19^^,^^20^^,^^21^^,^^22^ One of the mechanisms relating to the protective effect of PA against sarcopenia is the ability of PA to minimize the effects of muscle apoptosis, which causes decreases in the number and size of muscle fibers.^10^^,^^19^^,^^20^^,^^21^^,^^22^ In addition, sarcopenia is associated with falls and fractures among older adults, with elevated risk of mortality, given the effect of vitamin D on muscle strength and physical performance, which depend on the PA level demonstrated by elderly people.^4^^,^^23^

Nevertheless, only a few studies have focused on the relationship between sarcopenia and physical activity levels in the geriatric population, especially with regard to clinical variables relating to the diagnosis of sarcopenia, such as physical performance, handgrip strength and balance. In a cross-sectional study on community-dwelling older adults in Korea, it was found that moderate-to-vigorous physical activity was associated with a reduced risk of sarcopenia.^24^ In another study, it was observed that a higher level of PA (accelerometer-determined) was associated with greater lean mass percentage and greater lower-limb strength.^25^ However, contradictorily, other studies did not show any association between light/moderate/vigorous physical activity and sarcopenia.^26^ Moreover, physical activity (light/moderate/vigorous) did not seem to prevent the loss of muscle mass.^27^

There thus seems to be an issue that needs to be resolved regarding inconsistent findings relating to the effect of general physical activity on sarcopenia. Furthermore, only a few studies have examined the incidence rate of sarcopenia,^28^^,^^29^^,^^30^ of which only one study looked at its incidence in relation to physical activity.^28^ In addition, a few studies have used dual-energy X-ray absorptiometry (DXA) or bioelectrical impedance analysis to assess muscle mass.^28^^,^^29^^,^^30^

The differential that was proposed for the present study was that it should characterize physical activity levels and their association with clinical variables (using a simple and reliable questionnaire) through which the diagnosis of sarcopenia could become established. Thus, DXA, physical performance and handgrip strength and their relationship with energy expenditure would be investigated, following the recommendations of the European Working Group on Sarcopenia in Older People (EWGSOP). Furthermore, perceptions of the risk of falling, a critical factor for prevention of fractures and consequently mortality among elderly women, would be assessed. Better understanding of these factors might promote improvements in rehabilitation processes through activity and exercise, thereby creating strategies that would, in fact, be pragmatic with regard to prevention or treatment of sarcopenia due to advancing age.

## OBJECTIVE

The objective of the present study was to ascertain the effects of different levels of physical activity practice among elderly women on their clinical characteristics, physical performance, sarcopenia, handgrip strength and perception of the risk of falling, and the relationships of these factors with energy expenditure. The hypothesis was that elderly women who practiced vigorous physical activity would effectively gain better physical performance, thus improving their sarcopenia, handgrip strength and perception of the risk of falling, which might be related to higher energy expenditure.

## METHODS

### Study design and participants

This study had a cross-sectional and observational design. Fifty elderly women who were in care at the Beneficent Society for Healthcare for the Elderly of the Southern Region, São Paulo, Brazil, were evaluated, but three of them were excluded from the study because they did not attend the DXA examination. The study was approved by the Departmental Research Committee of the Universidade de Santo Amaro (UNISA) (registration number: 2.991.135; date: October 10, 2018, in accordance with relevant guidelines and regulations. All the elderly women provided their informed consent in writing before they underwent assessment.

The eligibility criteria for this study were that the participants needed to be elderly people practicing PA, aged 60 years or older. The exclusion criteria comprised presentation of vestibulocochlear diseases, uncontrolled cardiac and/or respiratory arrhythmias, convulsive and neurological syndromes and musculoskeletal disorders such as diabetic neuropathies, osteoarthritis, rheumatoid arthritis, limiting tissue lesions (cutaneous ulcers of any etiology) and functional impairment, especially of the lower limbs. In addition, use of prostheses and/or orthoses in the lower limbs, or occurrence of fractures within the last six months, were also considered to be exclusion criteria. Thus, good general health (without any diagnosis of mental illness or signs and symptoms of depression) was necessary in order to prevent bias in interpreting the evaluations.

The elderly women selected were divided into three groups: low PA: n = 13; moderate PA: n = 16; and high PA: n = 18. The PA level was measured by means of the International Physical Activity Questionnaire (IPAQ). IPAQ facilitates estimation of time spent walking, engaging in moderate and vigorous intensity PA, or sitting, both during the week and on weekends. It covers multiple domains: work, travel, housekeeping and leisure in one typical week or the last seven days. Detailed information on duration (minutes/day) and frequency (days/week) was collected for the different dimensions of physical and sedentary activity in all domains. In this, the activities performed for at least ten continuous minutes in the previous week were considered. Intensity, expressed in terms of working metabolic equivalents (METs), was determined in accordance with the guidelines for data processing and analysis that form part of IPAQ.^31^^,^^32^

To calculate PA scores in the different domains mentioned above, the following formula was used: Intensity (METs) * Duration (minutes/day) * Frequency (days/week). The total PA score was generated as the sum of the scores (work + travel + domestic + leisure) in METs/minute/week and the final classification of PA levels was obtained using the SAS statistical software, version 9.1 (SAS, Raleigh, NC, United States).

### Evaluation and clinical diagnosis of sarcopenia

The clinical and laboratory diagnosis of the skeletal muscle mass index (i.e. presence of sarcopenia) was made by means of lunar DXA examination (model DPX-MD; General Electric Company, United States), in order to characterize the sarcopenia profile of the elderly women. The parameters considered for the diagnosis of sarcopenia were ≤ 7.0 kg/m^2^ for men and ≤ 6.0 kg/m^2^ for women.^6^^,^^33^ This calculation was performed by means of the software inserted into the device and was called the skeletal muscle mass index. It was defined as the sum of the appendicular lean mass (as a volume) in the arm and leg segments, divided by the height squared.

### Physical performance and balance evaluation

Physical performance was determined using the Timed Up and Go (TUG) test, which provides an assessment of gait speed and dynamic balance. The TUG test measures the time spent in the tasks of standing up from a chair (from a seated position), walking three meters along a path marked out on the floor, turning round, walking along the same path back to the chair and sitting down again, with one’s back resting against the back of the chair.

The test began with the subject sitting correctly on a stable chair that had armrests and a seat back (the subject’s hips and back were leaning fully on the chair); the subject could use the arms of the chair to help in moving from the sitting position to the standing position and vice versa. At a signal from the evaluator (who started the timer concomitantly), the subject stood up, walked (at his or her usual pace) to a marked point, walked around it, returned and sat down on the chair again (the timer was stopped when he or she was in the correct sitting position, with arms resting on the chair armrests, at the end of the walk). The instruction given was for the elderly person to perform the task safely, as quickly as possible, wearing their usual shoes. For greater measurement accuracy, no bracing device for walking (walking stick, walker, etc.) or assistance from another person during the test course was allowed.^33^^,^^34^

The test results were classified as follows. Times of between 11 and 20 seconds were considered to be normal for frail elderly or disabled patients. Times that were greater than or equal to 20 seconds were taken to indicate impaired physical and balance performance that required appropriate intervention.^33^^,^^34^

### Handgrip strength assessment

Handgrip strength, in kg, was measured using a digital dynamometer (Model SH5001; Saehan Corporation, Yangdeok-Dong, Masan, South Korea). The test was performed with the elderly person sitting on a chair without armrests, with the shoulder slightly adduced and the elbow of the dominant arm flexed at 90°, and with the forearm and wrist in a neutral position. The elderly individual was instructed to press the dynamometer as hard as possible twice, with a two-minute interval between the attempts. The highest force value obtained was recorded. Elderly individuals with values below 30 and 20 (kg), for men and women respectively, were classified as having low muscle strength.^35^^,^^36^

### Fall risk assessment

The FRAQ-Brazil (Fall Risk Awareness Questionnaire) is a questionnaire that assesses the perception of risk of falling among individuals over 65 years of age, which has been validated for Brazilian culture.^37^ The questionnaire contains 26 objective multiple-choice questions and two open-ended questions, and is divided into two sections. The first section, with three questions, is applied by the interviewer him/herself, and the second section, with 25 questions, is answered individually by the interviewee him/herself. Each of the 26 multiple choice questions has only one correct answer. Because one question about medication contains eight correct answers and another question does not include feedback, the questionnaire scores can range from 0 to 32 points. The higher the number of points is, the better the elderly person’s perception of the risk of falling will be.^37^

### Statistical analysis

The sample-size calculations to determine that the sample should comprise 50 elderly people were based on an equation for the correlation coefficient (between the DXA test and MET, obtained for studying PA). A moderate effect size (F = 0.25), 80% power and a 5% significance level were used in the calculation. However, we ended up with a total of only 47 participants, since three elderly women who had previously been evaluated did not show up on the day and time scheduled for the DXA test. The groups of PA levels were compared for each of the dependent variables by means of analysis of variance, followed by Tukey’s post-hoc test. Multiple linear regression analysis was performed, considering the level of energy expenditure from the IPAQ (MET/min/week) in relation to the dependent variables evaluated, with a significance level of 5%. The statistical analysis was performed using SPSS v17.0 (SPSS, Chicago, IL, United States).

## RESULTS

The elderly people who were evaluated were similar regarding their anthropomorphic characteristics, between the different levels of PA considered, as shown in Table 1.

**Table 1.** Means and standard deviations of the elderly women’s anthropometric characteristics, compared between their levels of physical activity (PA): low, moderate or high

Anthropometric variables	Low PA (n = 13)	Moderate PA (n = 16)	High PA (n = 18)	P
Age (years)	73.4 ± 7.8	72.9 ± 5.4	69.9 ± 7.3	0.349
Height (m)	1.5 ± 0.7	1.5 ± 0.8	1.5 ± 0.5	0.130
Body mass (kg)	64.3 ± 11.6	71.5 ± 14.2	62.9 ± 9.9	0.112
Body mass index (kg/m^2^)	28.3 ± 3.9	28.9 ± 5.3	27.6 ± 4.3	0.739
Lean mass (kg)	35.6 ± 5.5	40.5 ± 8.2	36.8 ± 5.0	0.088
Fat mass (kg)	26.6 ± 7.6	28.6 ± 10.0	23.9 ± 7.0	0.353
Arterial hypertension	58%	50%	42%	-
Diabetes mellitus	25%	23%	14%	-

Analysis of variance test, with Tukey’s post-hoc test. P < 0.05 was taken to indicate a statistical difference (but there were no significant results).

Table 2 shows that the sarcopenia index at the high PA level indicated greater skeletal muscle mass than at the moderate and low PA levels. Another important observation, in relation to the IPAQ score, was that the energy expenditure and physical performance of the elderly people practicing high PA produced increases in these variables, compared with the elderly people practicing moderate and low PA. Regarding the other clinical variables, i.e. the perception of risk of falls and handgrip strength at different levels of PA, no significant differences were observed, as shown in Table 2.

**Table 2.** Means and standard deviations from the skeletal muscle mass index, Timed Up and Go (TUG) test, Fall Risk Awareness Questionnaire (FRAQ) and handgrip strength evaluations of the elderly women, compared between their levels of physical activity (PA): low, moderate or high

Clinical variables	Low PA (n = 13)	Moderate PA (n = 16)	High PA (n = 18)	P
IPAQ score (MET/min/wk)	840.8 ± 778.5	4788.0 ± 1906.9	5289.0 ± 2742.5	0.990^1-2^
				0.010^1-3*^
				0.266^2-3^
Skeletal muscle mass index (sarcopenia) (kg/m^2^)	5.7 ± 0.6	6.0 ± 0.4	6.3 ± 0.3	0.245^1-2^
				0.010^1-3*^
				0.007^2-3*^
Timed Up and Go (TUG) test (seconds)	12.2 ± 1.8	12.0 ± 4.2	13.3 ± 2.4	0.071^1-2^
				0.042^1-3*^
				0.086^2-3^
Fall Risk Perception Questionnaire (FRAQ) (score)	20.0 ± 3.6	21.3 ± 2.8	21.0 ± 2.5	0.341^1-2^
				0.212^1-3^
				0.102^2-3^
Handgrip strength (kg)	15.7 ± 3.7	17.1 ± 7.1	17.8 ± 4.8	0.153^1-2^
				0.357^1-3^
				0.099^2-3^

MET = working metabolic equivalent; min = minutes; wk = week.

Analysis of variance test, with Tukey’s post-hoc test. ^*^P < 0.05 was taken to indicate a statistical difference.

In the multiple linear regression analysis, it was observed that only physical performance and the perception of the risk of falling showed strong relationships with higher IPAQ score (MET/min/week), corresponding to moderate or high PA levels (Table 3). In addition, there was no positive relationship between IPAQ energy expenditure and the sarcopenia index and/or handgrip strength (Table 3).

**Table 3.** Multiple linear regression analysis on IPAQ energy expenditure (MET/min/week) at different levels of physical activity (PA) (low, moderate or high), in relation to the skeletal muscle mass index (sarcopenia), Timed Up and Go (TUG) test, Fall Risk Awareness Questionnaire (FRAQ), and handgrip strength evaluations among the elderly women

Clinical variables	Physical activity	Beta coefficient	Confidence interval (95%)	Standard deviation	T	P	R; R2
Skeletal muscle mass index (sarcopenia) (kg/m^2^)	Low	0.650	5.33; 6.06	0.6	1.7	0.102	0.42; 0.12
	Moderate	-0.221	5.79; 6.21	0.4	-0.4	0.693	0.10; -0.05
	High	0.595	6.15; 6.44	0.3	0.4	0.652	0.13; -0.07
Timed Up and Go (TUG) test (seconds)	Low	-0.329	11.10; 13.28	1.8	-1.7	0.101	0.42; 0.13
	Moderate	-0.293	9.76; 14.24	-4.2	-3.4	0.003^*^	0.65; 0.39
	High	-0.762	12.10; 14.49	2.4	-1.5	0.007^*^	0.41; 0.10
Fall Risk Perception Questionnaire (FRAQ) (score)	Low	0.770	17.82; 22.18	3.6	0.7	0.994	0.02; -0.07
	Moderate	0.148	19.80; 22.79	2.8	2.6	0.016^*^	0.55; 0.26
	High	0.417	19.76; 23.24	2.5	1.9	0.007^*^	0.41; 0.09
Handgrip strength (kg)	Low	0.690	13.46; 17.93	3.7	0.6	0.516	0.17; -0.03
	Moderate	-0.145	13.31; 20.88	7.1	-0.8	0.385	0.21; -0.01
	High	-0.762	15.41; 20.18	4.8	-1.52	0.156	0.42; 0.10

Multiple linear regression; ^*^P < 0.05 was taken to indicate a statistical difference.

T = t-statistic, calculated as t* = (sample coefficient - hypothesized value)/standard error of coefficient; R; R^2^ = coefficient of correlation; coefficient of determination.

## DISCUSSION

The main result from this study was that the loss of skeletal muscle mass (as measured using the sarcopenia index) in the high PA level group was lower than in the moderate and low PA level groups. Thus, the skeletal muscle mass of this group of elderly women with high PA was greater. Another important point was that the energy expenditure and physical performance were greater among the elderly women practicing high PA, compared with the moderate and low PA level groups. A further extremely valuable finding was the relationship between higher energy expenditure (as shown through the IPAQ), which corresponded to high and moderate PA levels practiced by these elderly subjects, and better perception of the risk of falling and increased physical performance.

The differential and clinical relevance of the present study was that it showed that practicing PA at different levels influenced the sarcopenia index of the elderly women evaluated, as measured through the DXA laboratory examination. A high PA level corresponded to an increased skeletal muscle mass index, in comparison with the groups with low and moderate PA levels. Current research suggests that, in particular, practicing PA is an effective supportive intervention for retarding reduction of muscle mass and strength in the elderly and, in addition, for promoting improvement in musculoskeletal functioning while performing activities of daily life.^14^^,^^19^^,^^20^^,^^21^^,^^22^

In the literature, a recent systematic review showed that individuals who were considered to be physically active presented lower risk of developing sarcopenia during the aging process,^38^ but it did not provide any description of the associated level of PA. In the present study, it was observed that, to prevent or alleviate the onset of sarcopenia, a high level of PA practice was necessary. This finding is especially relevant for the elderly in Brazil, where there is a lack of studies on the influence of different levels of PA practice among elderly women for preventing muscle loss.

Another important finding was that the energy expenditure verified through the IPAQ (MET/minute/week) for the high PA level group was greater, with better physical performance. This can be explained by the effect of the intensity of high PA practice on improving the aerobic conditioning of the elderly. This shows the importance of increasing the PA intensity for prevention of sarcopenia and the risk of falling, in view of the positive association between sarcopenia and falls and fractures among older adults.^38^^,^^39^^,^^40^^,^^41^ The findings from the present study were concordant with those of some reviews and clinical studies, in which the effectiveness of aerobic exercise of 30 to 60 minutes for preventing the risk of dynapenia, sarcopenia, and associated weaknesses in the elderly was revealed.^2^^,^^4^^,^^5^^,^^39^ In addition, it has also been shown in the literature that prolonged periods of sedentary behavior among the elderly do not substantially increase energy expenditure above resting levels (< 1.5 units of metabolic work equivalent, METs). This leads to impaired muscle functioning^40^^,^^42^ and performance in the elderly.^41^^,^^42^^,^^43^

In this study, all the elderly people performed aerobic exercises, resistance exercises and walking as their PA practice. Only those who were considered to have high PA levels were able to improve their muscle mass loss and physical performance. This was concordant with the findings of Santos et al., who showed that insufficient leisure-time PA practice was associated with sarcopenia in individuals aged 50 years or over.^14^ Although those authors did not evaluate elderly people, the results from the present study emphasize the effectiveness of high PA levels for prevention of sarcopenia. This finding with regard to higher intensity of PA, in association with anaerobic exercise (i.e. for 60 minutes, twice a week), was also observed in some other studies,^2^^,^^39^^,^^40^ thus confirming the association of these two factors with long-term improvement of sarcopenia.

Another extremely valuable finding was that physical performance and the perception of the risk of falling had positive relationships with the IPAQ energy expenditure consumption (MET/minute/week), which corresponded to moderate and high PA levels practiced by the elderly women. On the other hand, Aggio et al. did not find any association or relationships between sedentary patterns and sarcopenia among men aged 70 to 92 years.^44^ In the present study, only elderly women were evaluated and it was possible to establish a cause-effect relationship between increased energy expenditure (moderate and high PA levels) and better perception of the risk of falling and increased physical performance among them. These relationships support development of pragmatic and effective strategies for prevention of falls and their consequences among elderly women. These consequences include higher numbers of hospitalizations, which generate high healthcare costs and high mortality rates resulting from femur fractures, and decreased PA resulting from restrictions on its practice.^38^^,^^39^^,^^40^^,^^41^^,^^44^^,^^45^

Through gaining an understanding that moderate or high PA is related to better physical performance and perception of the risk of falling, elderly people’s fear of falling may be reduced. It may help combat loss of independence and the ability to perform activities of daily living normally, which arise through limitations on practicing PA.^26^^,^^27^^,^^30^^,^^46^ Scientific evidence has increasingly shown that practicing PA is very effective in preventing falls, since it increases muscle strength and improves balance, flexibility, motor coordination and proprioception.^2^^,^^4^^,^^14^^,^^46^

The contribution that this study has made to was to show that moderate and high levels of PA made a difference through improving the perception of the risk of falling, and thus through maintaining elderly people’s perceptive self-care for better fall prevention. The practical implications of this study are the following:

- High physical activity levels gave rise to increases in the skeletal muscle mass index and physical performance. Poor levels of these clinical characteristics are associated to sarcopenia in elderly women;
- High and moderate physical activity levels (energy expenditure in MET/minute/week) were a good predictor of improved physical performance and better perception of the risk of falling among elderly women;
- Public health interventions aimed at encouraging moderate and high levels of physical activity may increase physical performance and reduce the risk of falling, thus reducing the onset of sarcopenia in elderly women.

The limitation of this study was that it did not evaluate the dynamic balance between the levels of PA and sarcopenia, to better understand falls among the elderly people evaluated here. Future studies addressing variables relating to kinematic and dynamic balance, according to the PA levels of elderly people as they age, may further enhance the understanding of the association of these variables with the sarcopenia index and with falls.

## CONCLUSION

Elderly women classified as presenting high levels of PA showed improvements in sarcopenia, handgrip strength, physical performance and perception of the risk of falling. The IPAQ energy expenditure of the elderly women with high and moderate PA levels was a good predictor of physical performance and of improved perception of the risk of falling.
